# Supplementary material for: The Motion Paradigm of Pre-Dock Zearalenone Hydrolase Predictions with Molecular Dynamics and the Docking Phase with Umbrella Sampling
Source: Molecules. 2023 Jun 4;28(11):4545. doi: 10.3390/molecules28114545 (PMC10254720; doi:10.3390/molecules28114545)
Supplement: Supplementary file 1 [file molecules-28-04545-s001.zip › SUPPLEMENTARY INFORMATION.pdf]

Supplemental Materials

Title:

The Motion Paradigm of Pre-dock Zearalenone Hydrolase Predicts with Molecular Dynamic and the Docking One with Umbrella Sampling

Author:

Xi-Zhi Hong[1], Zheng-Gang Han[1], Jiang-Ke Yang[1*], Yi-Han Liu[2*]

[1]Pilot Base of Food Microbial Resources Utilization of Hubei Province, College of Life Science and Technology, Wuhan Polytechnic University, Wuhan, China

[2]Key Laboratory of Industrial Fermentation Microbiology, Ministry of Education, The College of Biotechnology, Tianjin University of Science and Technology, Tianjin, China

*** Correspondence:**

Jiang-Ke Yang

yang.jiangke@whpu.edu.cn

Yi-Han Liu

lyh@tust.edu.cn

Figure legend

Figure S1. Violin plot describe the number of representative sequences at different identity. The number at 60%, 70%, 80%, 90%, and 95% identity were plotted.

Figure S2. E_{MM} calculation of mutant D45A and R139A compares to the wild type. (A)-(B) E_{MM} of the complex with mutants D45A and R139A. (C)-(D) E_{MM} of the interaction of the NbZHD and the zearalenone with mutants D45A and R139A.

Figure S3. RMSD of the concatenated window simulation. The concatenated simulation consists of 3000 frames and each frame is separated by 2 ps.

Figure S4. The structure colored smudge is the state 2 whose D170-R190 are colored slate. The structure colored smudge is the state 2 whose D170-R190 are colored slate. The structure colored wheat is the unbound state whose D170-R190 are colored violetpurple. The structure colored teal is the ZHD101 whose V153-H173 are colored olive. The structure colored gray is the ZHD607 whose V156-A176 are colored red. (A) Structure discrimination of the bolt between the state before ligand-docking of NbZHD and State 2. (B) Structure discrimination of the bolt between the state before ligand-docking of NbZHD and ZHD101. (C) Structure discrimination of the bolt between the state before ligand-docking of NbZHD and ZHD607. (D) Structure discrimination of the bolt between ZHD607 and ZHD101.

Video S1. The ligand rotates from the docking state to the releasing state.

Video S2. The ligand is released from the aperture of the hydrolase domain at the side view.

Video S3. The ligand is released from the aperture of the hydrolase domain at the front view.

Table S1. The table presents the frequency of occurrence of different residues within the 384 sequences involved in the motion pathway.

Figure S1

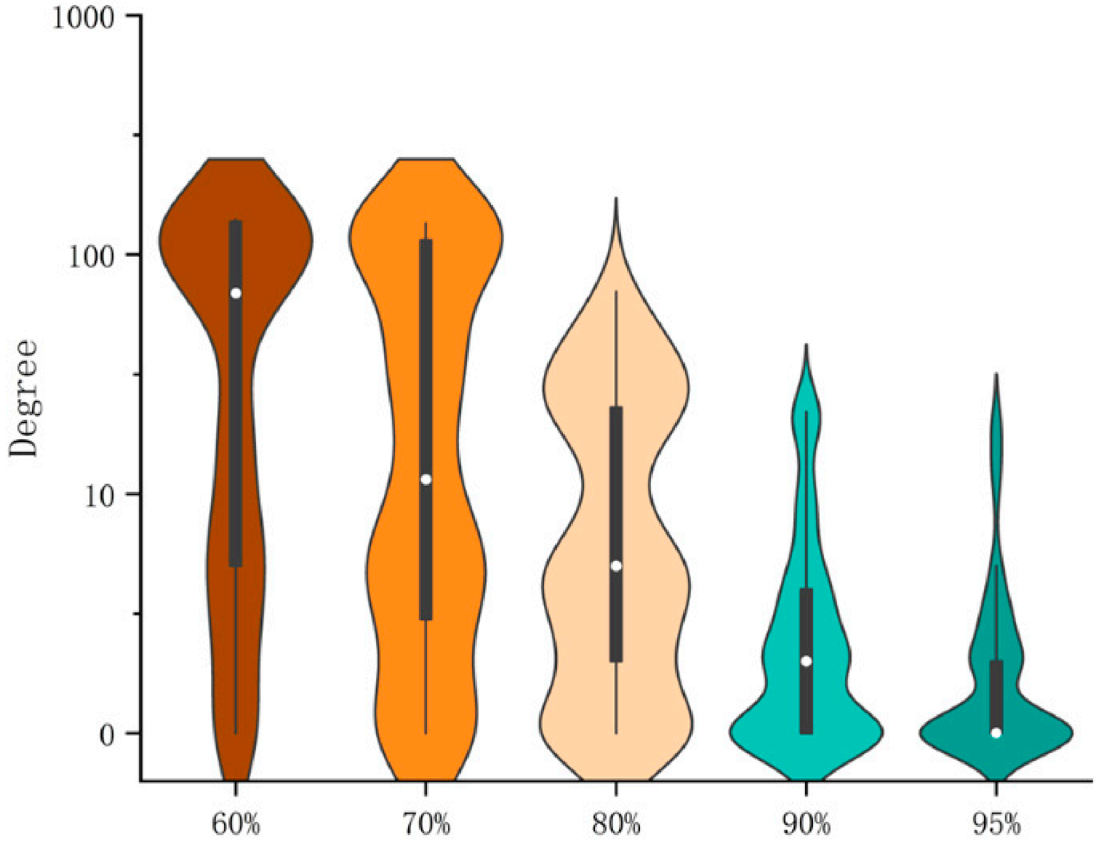

Figure S2

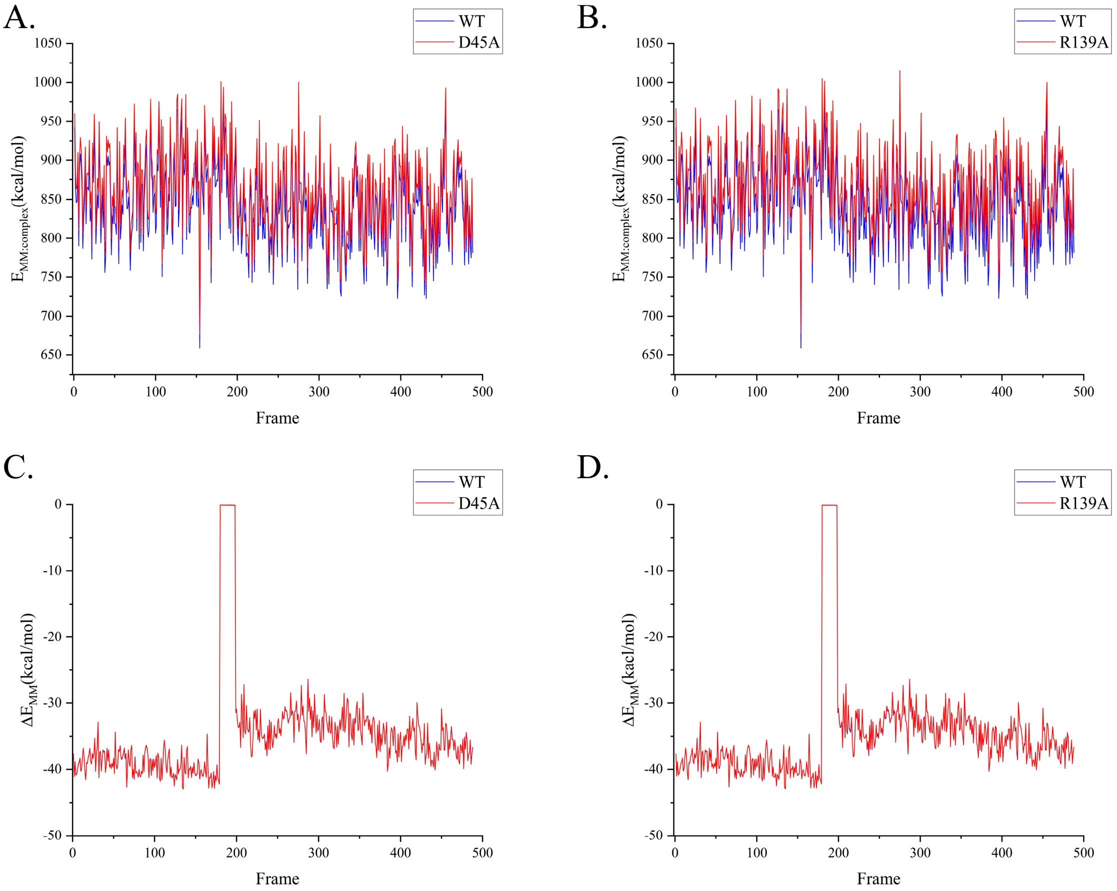
A.
WT
D45A
E_MM:complex(kcal/mol)
Frame
B.
WT
R139A
E_MM:complex(kcal/mol)
Frame
C.
WT
D45A
ΔE_MM(kcal/mol)
Frame
D.
WT
R139A
ΔE_MM(kacl/mol)
Frame

Figure S3

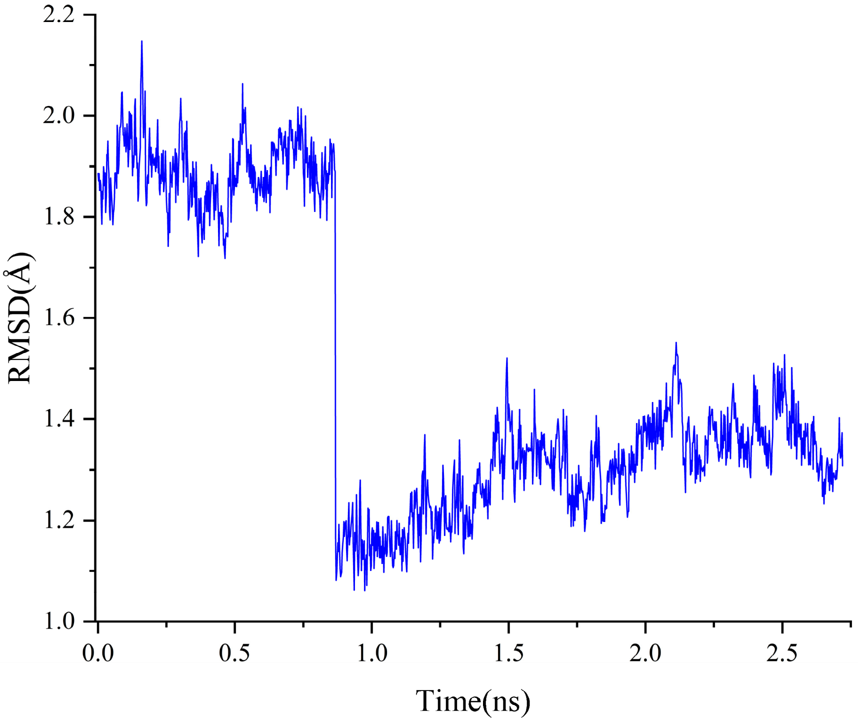

Figure S4

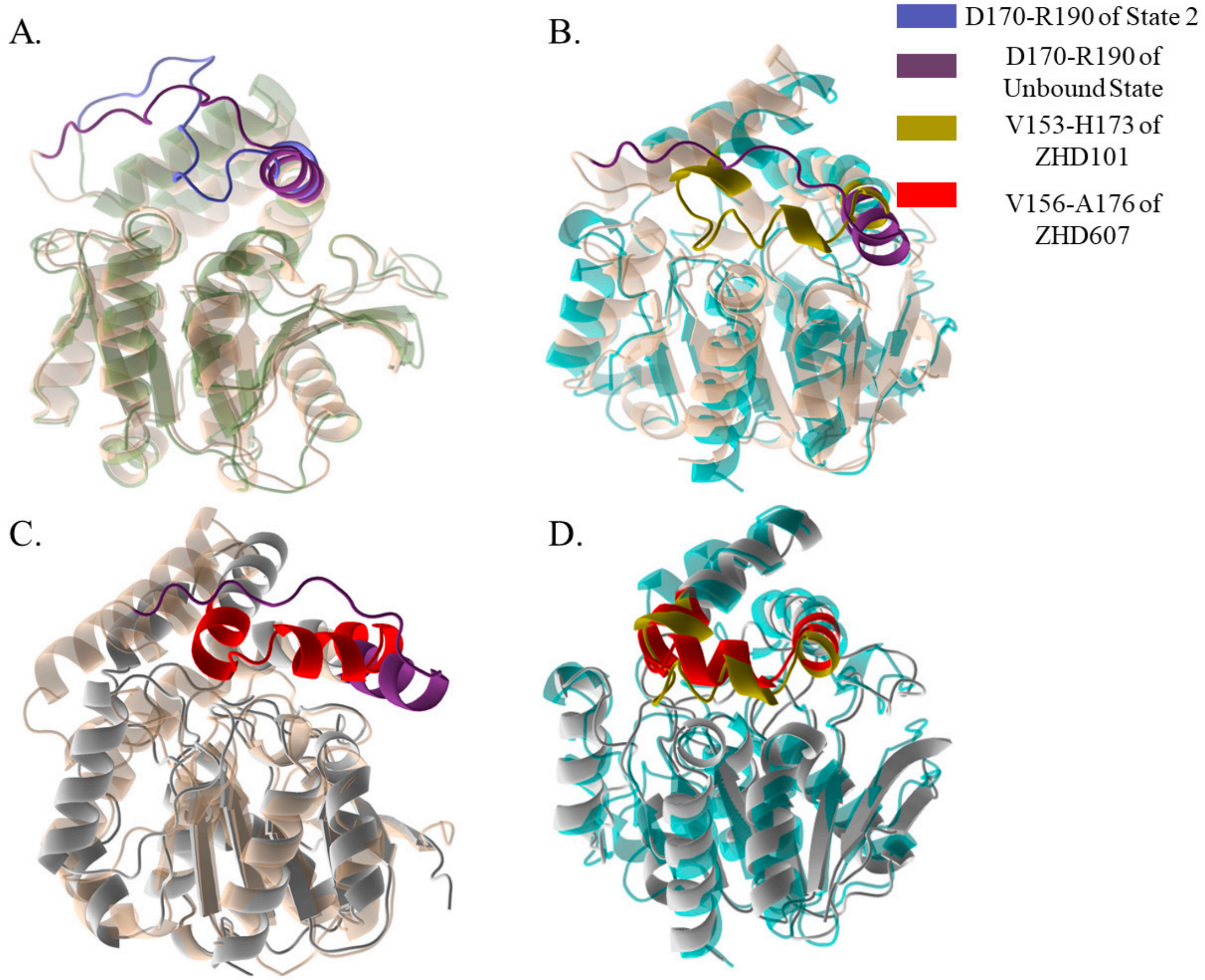
A.
B.
C.
D.
D170-R190 of State 2
D170-R190 of Unbound State
V153-H173 of ZHD101
V156-A176 of ZHD607

Table S1

	38	45	62	66	139	145	146	149	152	154	174	177	183	202	207
-	0	0	0	3	4	2	20	11	24	1	26	17	19	1	35
A	27	144	6	31	14	9	6	27	22	45	124	43	41	4	69
C	0	0	0	5	0	1	5	1	0	1	0	2	18	4	2
D	125	17	0	45	0	0	40	30	16	4	6	59	7	13	4
E	45	11	0	3	7	2	68	88	78	16	9	85	23	60	3
F	0	7	0	2	13	7	1	104	4	3	0	6	8	6	1
G	19	60	10	2	3	1	10	3	16	2	54	57	5	7	4
H	0	50	1	6	3	1	10	16	7	5	0	2	4	1	0
I	0	0	0	9	17	30	2	5	4	38	0	1	0	120	8
K	10	3	0	26	4	9	8	0	13	7	7	5	4	4	1
L	0	1	1	185	138	119	4	30	2	136	7	5	6	3	7
M	0	1	0	4	6	16	1	3	3	14	7	0	1	5	118
N	3	1	0	11	3	1	0	3	1	15	3	1	2	0	0
P	45	0	1	10	2	1	20	1	1	14	101	66	14	7	24
Q	4	60	0	3	2	4	88	1	72	2	8	9	88	1	3
R	15	9	0	8	151	4	54	2	17	2	11	5	32	1	6
S	76	8	359	12	1	2	6	2	84	7	8	7	8	14	55
T	15	10	5	2	7	1	8	2	5	14	9	2	12	105	40
V	0	1	1	15	7	167	4	2	11	56	2	12	56	27	4
W	0	0	0	2	1	6	0	0	0	0	0	0	2	0	0
Y	0	1	0	0	1	1	29	53	4	2	2	0	34	1	0
V_S*	1.733	1.900	4.272	2.178	2.311	2.336	1.338	1.610	1.426	1.733	1.845	1.438	1.195	1.867	1.655
Conserved Residues**	D	A	R	L	L/Q	T	E/P/Q	E/F	E/Q/S	L	A/P	D/E/G/P	Q/V	I/T	M
NbZHD***	A	D	S	L	R	V	Q	L	Q	L	P	E	Q	I	M

*: V_S is the coefficient of variation. When V_S is over 2, the corresponding sites are considered as highly conserved; while it below 2, the corresponding sites are considered as weakly conserved. And when V_S is over 1, the corresponding sites are considered conserved. **: The conservative residues were selected based on their high frequency of occurrence and those with less than 40 occurrences. ***: The corresponding residue in NbZHD whose motion pathway is been detected.
